# The Regulatory Role of MicroRNA in Hepatitis-B Virus-Associated Hepatocellular Carcinoma (HBV-HCC) Pathogenesis

**DOI:** 10.3390/cells8121504

**Published:** 2019-11-24

**Authors:** Kurt Sartorius, Julia Makarova, Benn Sartorius, Ping An, Cheryl Winkler, Anil Chuturgoon, Anna Kramvis

**Affiliations:** 1 Department of Public Health Medicine, School of Nursing and Public Health, University of KwaZulu-Natal, Durban 4041, South Africa; benn.sartorius1@lshtm.ac.uk (B.S.); chutur@ukzn.ac.za (A.C.); 2 Faculty of Commerce, Law and Management, University of the Witwatersrand, Johannesburg 2193, South Africa; 3 UKZN Gastrointestinal Cancer Research Centre, Durban 4041, South Africa; 4 Hertsen Moscow Oncology Research Institute, Branch of the National Medical Research Radiological Center, Ministry of Health of the Russian Federation, 125284 Moscow, Russia; j-makarova@yandex.ru; 5 Engelhardt Institute of Molecular Biology, Russian Academy of Sciences, 119991 Moscow, Russian; 6 Faculty of Biology and Biotechnologies, National Research University Higher School of Economics, 101000 Moscow, Russia; 7 Frederick National Laboratory, Basic Research Laboratory, Centre for Cancer Research, National Cancer Institute, Frederick, MD 21702, USA; ping.an@nih.gov (P.A.); winklerc@mail.nih.gov (C.W.); 8 Hepatitis Virus Diversity Research Unit, Department of Internal Medicine, School of Clinical Medicine, Faculty of Health Sciences, University of the Witwatersrand, Johannesburg 2193, South Africa; Anna.Kramvis@wits.ac.za

**Keywords:** regulatory, role, dysregulated, miRNA, hepatitis B infected hepatocellular carcinoma, HBV-HCC continuum

## Abstract

The incidence and mortality of hepatitis B virus (HBV)-associated hepatocellular carcinoma (HBV-HCC) is an intractable public health problem in developing countries that is compounded by limited early detection and therapeutic options. Despite the early promise of utilizing the regulatory role of miRNA in liver cancer, this field remains largely in the work-in-progress phase. This exploratory review paper adopts a broad focus in order to collate evidence of the regulatory role of miRNA in each stage of the HBV-HCC continuum. This includes the regulatory role of miRNA in early HBV infection, chronic inflammation, fibrosis/cirrhosis, and the onset of HCC. The paper specifically investigates HBV dysregulated miRNA that influence the expression of the host/HBV genome in HBV-HCC pathogenesis and fully acknowledges that this does not cover the full spectrum of dysregulated miRNA. The sheer number of dysregulated miRNA in each phase support a hypothesis that future therapeutic interventions will need to consider incorporating multiple miRNA panels.

## 1. Hepatitis B Virus-Associated Hepatocellular Carcinoma (HBV-HCC)

An estimated 841,080 new liver cancers (LCs) were diagnosed worldwide in 2018, with hepatocellular carcinoma (HCC), the most common histological subtype, accounting for 75% to 90% of LCs [1]. A 12.4% increase of LC was recorded between 2008 (696,000) to 2012 (782,000) [2] and [3] 7.6% between 2012 and 2018 (841,080) [1]. HCC is listed globally as a leading cancer in men and women [4], with a recent report indicating that 83% of new cases diagnosed occurred in less well developed countries in East and south Eastern Asia and Western and Central Africa [5] [GLOBOCAN 2018 (http://gco.iarc.fr), accessed on 28 October 2019].

HBV and hepatitis C virus (HCV) infection is linked with more than 90% of all HCC incidence in developing countries compared to 40% in developed countries, with HBV being the major contributor in 66% of the cases [6]. According to the estimates of the global burden of disease study, deaths due to viral hepatitis increased by 63%, from 0.89 million to 1.45 million, between 1990 and 2013 [7,8]. HBV has been coined as “the second most dangerous carcinogen after tobacco” [8,9]. HBV, the prototype member of the family *Hepadnaviridae*, is hepatotropic and non-cytopathic. HBV enters the hepatocyte via the sodium-taurocholate cotransporting polypeptide (NTCP) receptor [10] and replicates by reverse transcription of a RNA intermediate, the pregenomic RNA (pgRNA). Although not required for replication of HBV, integration of HBV DNA can occur within the host genome and this has been implicated in hepatocarcinogenesis [11].

### 1.1. HBV-HCC Pathogenesis Continuum

HBV infection can result in a cascade of complex interactions between the host and the virus, which can lead to a spectrum of clinical manifestations including the asymptomatic carrier state, acute or fulminant hepatitis, and, ultimately, chronic hepatitis with progression to HCC with or without intermediary cirrhosis [12]. In 95% of infected adults and in only 10% of children, acute infection is self-limiting, with the virus persisting in 5% and 90% of the cases, respectively [12,13], with the risk of HCC increasing 100-fold if a patient is positive for both the hepatitis B surface antigen (HBsAg) and the hepatitis B e antigen (HBeAg). Even in the absence of HBsAg, there is a reduced and yet significant risk of HCC [14]. As viral load increases, the host immune response, triggered by HBV surface antigens (HBsAg), elicit T-cell responses accompanied by secondary inflammatory response [14], and an increase in free radicals, interferon, tumor necrosis factor (TNF), and hepatic injury. Moreover, if integration of HBV DNA into the host hepatocyte genome occurs, it results in the oncogenic disruption of cellular genes [15]. This disruption triggers apoptosis, regeneration, and early senescence [16], in addition to host cell deletions, *cis*/*trans*-activation, translocations, the production of fusion transcripts, aberrant epigenetic changes, and generalized genomic instability [17]. In parallel with these changes, the continuous destruction of well-differentiated hepatocytes and organized extracellular matrix results in their eventual replacement with undifferentiated liver stem cells and poorly organized fibrotic tissue [12].

**Figure 1:**
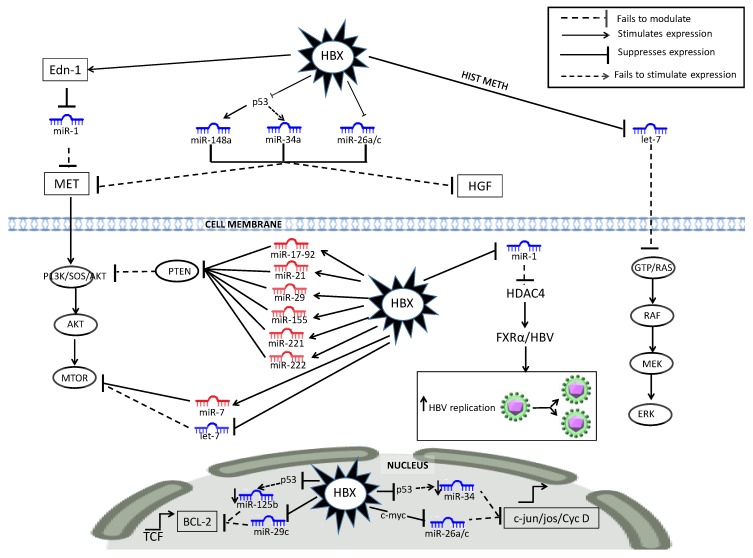
HBx-dysregulated miRNA in the P13K/MAPK liver cancer pathway. HBV-specific abbreviations include endothelin-1 (*Edn-1*), HIST METH (histone methylation), Farnesoid X receptor (*FXRα*).

**Figure 2:**
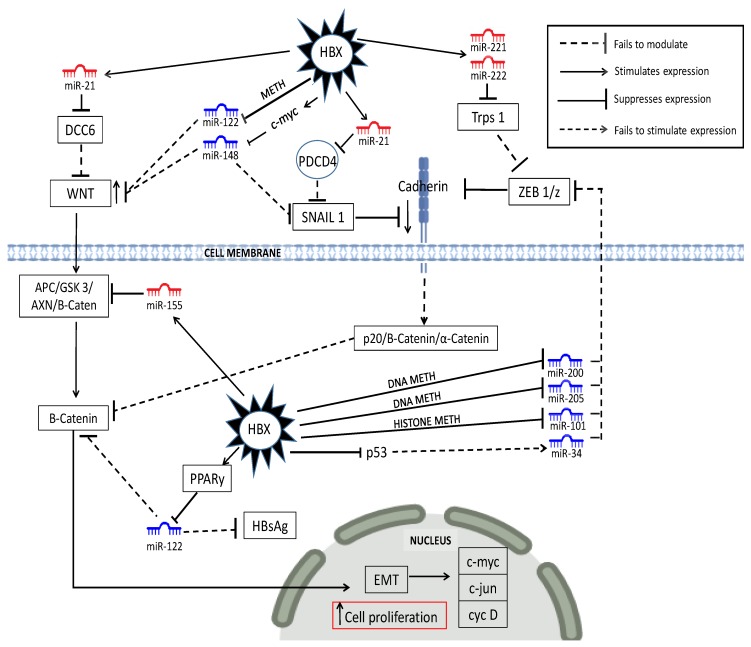
Dysregulated miRNA in the WNT/β-catenin liver cancer pathway. HBV-specific abbreviations DCC6 (DCC-adaptor protein), DNA METH (DNA methylation), HISTONE METH ((histone methylation), PPARγ (peroxisome proliferator-activated receptor gamma), EMT (epithelial mesenchymal transition).

**Figure 3:**
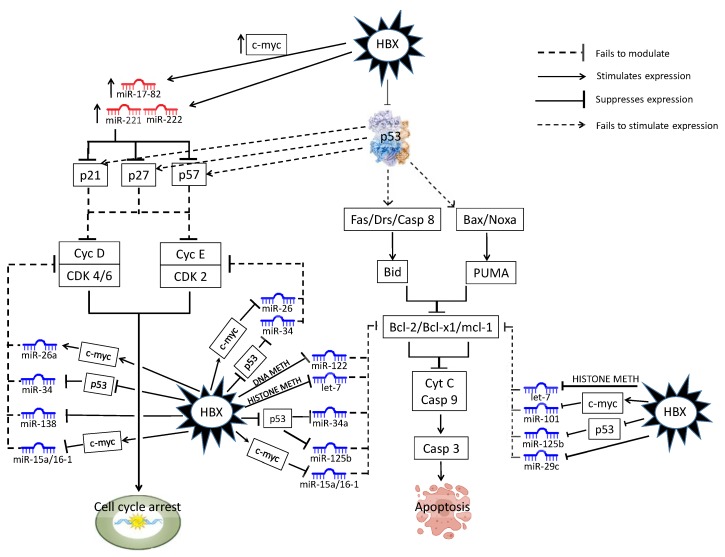
Dysregulated miRNA in the TP53 liver cancer pathway. HBV-specific abbreviations include DNA METH (DNA methylation), HISTONE METH (histone methylation).

**Figure 4:**
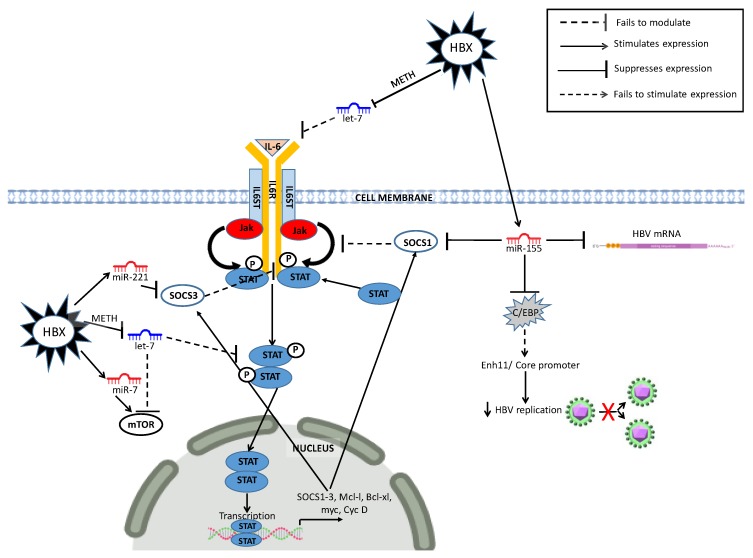
Dysregulated miRNAs in the JAK/STAT liver cancer pathway. METH (Methylation) C/EBP (CCAAT/enhancer-binding protein) Enh11 (enhancer 11).

HBV-HCC pathways typically include aberrant expression in the retinoblastoma-tumor protein 53 (RB1-TP53) suppressor networks, the Wingless-related integration site/beta-catenin (WNT/β-Catenin) pathway, and the phosphoinositide 3-kinase/mitogen-activated protein kinase (PI3K/MAPK) and Janus kinase/signal transducer (JAK/STAT) pathways [18,19] (see Figure 1, Figure 2, Figure 3 and Figure 4).

This paper specifically adopts a broad, exploratory review of the regulatory role of HBV dysregulated miRNA in the HBV-HCC continuum and miRNA dysregulation may provide a means of screening HBV carriers and developing HCC biomarkers and alternate therapeutic options.

### 1.2. The Deregulation of MiRNA in HBV-HCC Continuum

MicroRNA (miRNA) act as post-transcriptional gene silencers that collectively reduce or inhibit their target mRNA expression, thereby playing a homeostatic role that fine tunes the translation of proteins. The ancillary role of miRNA, as mild suppressors, has been explained by the inherently stochastic nature of gene transcription and environmental fluctuations [20]. In the case of transient reaction to environmental conditions, miRNA quantity become temporarily dysregulated until homeostasis is restored [21]. In the case of the continuum from asymptomatic HBV infection leading to HCC, multiple miRNA become increasingly permanently dysregulated (Table 1, Table 2, Table 3 and Table 4) as a result of HBV infection, epigenetic changes [22], inflammation [23], fibrosis [24], cirrhosis [16], and the onset of HCC. The increasing level of dysregulation in the HBV-HCC continuum is illustrated in a study that showed 37 miRNA deregulated in otherwise healthy controls (HC), 77 in asymptomatic HBV carriers (ASC), 101 in chronic hepatitis B infection (CHB) (Table 1), and 135 in acute liver failure (ALF) [25]. In general, dysregulated miRNA in the HBV-HCC continuum fall into two major categories, namely, those that target HBV transcripts and those that target the host cell genome expression.

## 2. MiRNA Dysregulation in the HBV-HCC Continuum

This section illustrates the regulatory role of HBV dysregulated miRNA in the different stages of the HBV-HCC continuum (Table 1, Table 2, Table 3 and Table 4). A brief description of each stage is provided before describing the regulatory role of miRNA.

### 2.1. Early/Chronic HBV Infection

In this stage the pivotal role of HBV, in cases where viral clearance does not occur, appears mainly to be due to the continuous destruction of hepatocytes by T-cells, which attempt to eliminate the infection in conditions of chronic inflammation and increasing oxidative stress [18]. If viral clearance does not occur, patients that progress to CHB will elicit a weak peripheral cytotoxic T-lymphocyte (CTL) response. Typically, an activated humoral response in CHB involves the production of interleukin-4 (IL-4), IL-5, and IL-10 secreted by type-2 helper T lymphocytes that promote antibody production rather than viral clearance. It is also hypothesized that low levels of intrahepatic HBV-specific CTLs are the cause of hepatic inflammation flares that occur in CHB patients without viral clearance [26]. Host cell miRNA modulate HBV expression by either targeting own cell transcription factors required for HBV genome transcription or by directly binding to HBV transcripts [13]. Many host cell miRNAs are also modulated by HBV proteins in order to ensure the persistence and replication of the virus.

#### MiRNA Regulating HBV Genome Expression

**Scheme 1:**
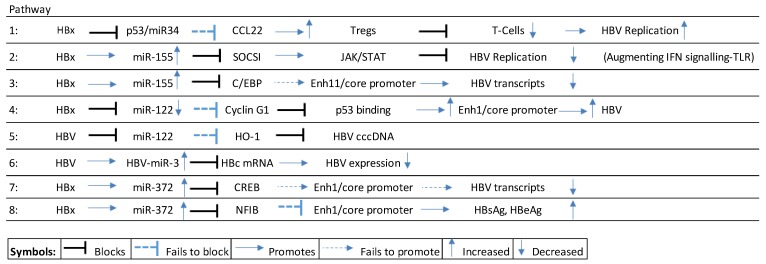
HBx-mediated miRNA pathways in early/chronic HBV infection. CCL22: C-C motif chemokine 22; SOCS1: Suppressor of cytokine signaling 1; C/EBP: CCAAT/enhancer-binding protein; HO1: heme oxygenase 1; CREB: c-AMP-response element binding protein.

HBV genome replication and survival is self-managed as a result of modulating (up/down) both host cell miRNA expression, as well as coding for its own miRNA [27]. The hepatitis B virus x protein (HBx), for example, blocks p53 stimulated miR-34 expression in hepatocytes leading to the upregulation of macrophage-derived chemokine (CCL22) that stimulates regulatory T-cells (Tregs) that, in turn, block effector T-cells allowing HBV expression to increase [28,29] HBV genome transcription or by directly binding to HBV transcripts [13] (see Scheme 1, pathway 1).

Upregulation of miR-155 by HBx results in the downregulation of suppressor of cytokine signaling-1 (SOCSI) expression contributing to increased JAK/STAT signaling, leading to the suppression of HBV infection by augmenting interferon (IFN) signaling [30] (see Scheme 1, pathway 2). Alternatively, HBx upregulated miR-155 also subdues HBV replication by blocking the CCAAT/enhancer-binding protein (C/EBP) that binds and activates the HBV Enhancer (Enh) 11/core promoter [31] (see Scheme 1, pathway 3).

The liver miR-122 is widely reported as upregulated in serum following HBV infection (references in Table 1) and is regarded as a key suppressor of HBV expression [32,33,34]. However, it has been reported that HBx downregulates miR-122 and facilitates increases in HBV transcripts by failing to block Cyclin G1, which then blocks p53 from binding to HBV Enh1/core promoter thus facilitating an increase in HBV expression [35] (see Scheme 1, pathway 4). Other studies have demonstrated that miR-122 blocks HBV pgRNA that encodes the hepatitis B core antigen (HBcAg) and viral polymerase, and that HBV downregulated miR-122 blocks HBV replication by failing to modulate heme-oxygenase (HO-1), which blocks HBV covalently closed circular DNA (cccDNA) [36] (see Scheme 1, pathway 5).

Multiple miRNA directly target HBV transcripts and examples of these include miR-184/-185/-196a/-199a-3p/-210/-217 that are all significantly upregulated [28]. Recently it was also discovered that the HBV virion generates its own HBV-miR-2/-3 [37]. HBV-miR-3 suppresses the hepatitis B core protein (HBc) mRNA to self-regulate HBV expression downwards [27], possibly in order to promote its survival by way of avoiding the host immune system [28,38] (see Scheme 1, pathway 6).

Some miRNA, like miR-372, can influence HBV expression positively and negatively depending on their target pathways. HBx upregulated miR-372 [39,40] targets the c-AMP-response element binding protein (CREB) from binding to HBV enhancer 1/core promoter (Enh 1/ENI-Cp) to reduce HBV transcripts. It also targets the nuclear factor 1 B-type protein (NF1B) that fails to modulate HBV Enh 1 (ENI-Cp), thus promoting HBV expression [28,39] (see Scheme 1, pathway 7–8).

### 2.2. HBV-Induced Inflammation Pathways

**Scheme 2:**
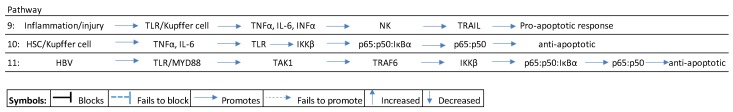
Inflammation pathways in HBV-induced pathways. HSC: hepatocyte stellate cell; TLR: toll-like receptor; TNF: tumor necrosis factor; NK: natural killer cells; TRAIL: TNF-related apoptosis-inducing ligand; TAK1: transforming growth factor beta-activated kinase 1; IKKβ: inhibitor of NFκB kinase.

In this stage, chronic inflammation, accompanied by T-cell response, causes tissue damage where the continuous replacement of hepatocytes is initially achieved by differentiated hepatocytes, which eventually become depleted and are replaced by the recruitment of non-epithelial cells in the liver [18]. While their activation and proliferation initially serves to support the immune response and the regeneration of the tissue, during chronic liver damage and with increasing inflammation, their expansion predominates and they gradually replace epithelial structures in the organ. Inflammation/injury induces pro-inflammatory cytokines like tumor-necrosis factor-alpha (TNFα) and interleukin 6 (IL-6) by activating Kupffer cells and liver-derived macrophages. The activation of pro-inflammatory cytokines like TNFα and Il-6 can induce both pro-apoptotic and anti-apoptotic effects in injured liver tissue [41,42]. A pro-apoptotic response would, typically, be triggered by inflammation/necrosis that triggers Toll-like receptor (TLR) signaling to induce Kupffer cells to synthesize pro-inflammatory cytokines like TNFα, IL-6, and interferon alpha (IFNα), that recruit natural killer cells (NKs) to promote TNF-related apoptosis-inducing ligand (TRAIL) [43,44,45] (see Scheme 2, pathway 9).

Alternatively, an anti-apoptotic response results from the activation of the canonical nuclear factor-κB (NFκB) inflammation pathway [46]. Injury triggers HSC/Kupffer cell stimulation of chemokines like TNFα and IL-6 that induce TLR signaling in hepatocytes. TLR signaling then activates the inhibitor of kappa kinase (IKK) complex to phosphorylate the IαKα/p65:p50 bound complex in the cytoplasm that leads to the accumulation of transcription factors p65:p50 in the nucleus. This accumulation, in turn, promotes an anti-apoptotic response [41,47,48] (see Scheme 2 pathway 10).

In CHB, a deficiency in interferon can block the immune response by subduing TLR expression, and the HBx protein can stimulate TGF-β1 to induce T-cell regulators to subdue the immune response [49,50]. Persistent activation of the NFκB pathway in HBV infection is orchestrated by way of TLRs that bind to HBV proteins (e.g., HBx) and activate the NFκB pathway. Typically HBV proteins bind to TLR-4/7/9 and recruit the myeloid differentiation primary response adaptor protein (MYD88) to activate tumor necrosis factor receptor (TNF-R)-associated factor 6 (TRAF6) via transforming growth factor beta-activated kinase 1 (TAK1), to activate the inhibitor of nuclear factor kappa-B kinase (IKK complex), to induce nuclear translocation of p65:p50 accumulation in the nucleus, and this results in an anti-apoptotic effect [41,49,51] (see Scheme 2 pathway 11).

#### MiRNA Regulating HBV-Induced Inflammation Pathways

At the commencement of the inflammation–fibrosis continuum in persistent HBV infection, a wide range of host miRNA are deregulated (see Table 1). Many of these miRNA subtly manipulate the host immune response to ensure both the survival and proliferation of the virus [28]. Complex interactions between HBV, the immune system, and miRNA trigger TLR pathways that interact with cytokines, interferons, and TNFs to modulate innate immune responses [51].

**Scheme 3:**
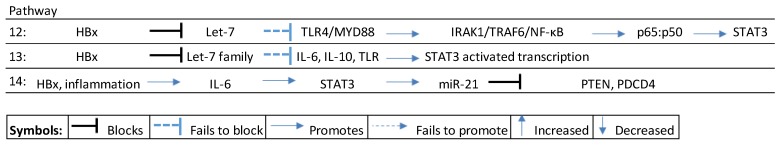
HBx-induced miRNA in inflammation pathways. TLR: toll-like receptor; IRAK1: interleukin-1 receptor associated kinase 1; TRAF6: tumor necrosis factor receptor associated factor 6; IL: interleukein; STAT3: signal transducer and activator of transcription 3; PTEN: phosphatase and tensin gene; PDCD4: programmed cell death protein 4 gene.

Examples of miRNA-influenced inflammation include miR-145/-148a/-200b/-200c/-455 and the Let-7 family [51]. Typically the Let-7 family, for instance, is downregulated in CHB by the HBx protein (or alternatively “mopped” up by HBV mRNA), thus preventing it from suppressing TLR4 signaling along the interleukin-1 receptor associated kinase (IRAK1)/TRAF6/NF_K_B pathway, to induce nuclear accumulation of p65:p50 [51] that promotes STAT3, resulting in cell proliferation and survival [48,52] (see Scheme 3, pathway 12). Alternatively, HBx downregulates Let-7 that can also activate STAT3 signaling by failing to modulate the expression of inflammatory cytokines, to influence miRNA-mediated suppression of key tumor suppressors [53] (see Scheme 3, pathway 13). HBx-induced upregulation of miR-21 by Il-6 mediated STAT3 signaling typically blocks tumor suppressors like *PTEN* and *PDCD4* at an early stage in the inflammation–fibrosis axis and continues in the HBV-HCC stage [54,55,56] (see Scheme 3, pathway 14).

### 2.3. MiRNA Regulating HBV-Induced Fibrosis/Cirrhosis

In this stage, chronic tissue damage and inflammation are accompanied by the activation of non-epithelial cells in the liver, which proliferate. Initially this activation supports the immune response to regenerate damaged tissue, as these non-epithelial cells gradually replace epithelial structures in the liver. This process; however, eventually manifests as fibrosis/cirrhosis where well-organized parenchymal tissue is increasingly replaced by disorganized and dysfunctional fibrotic tissue [18]. In the inflammation–fibrosis axis, fibrogenesis is orchestrated by a complex network of common cytokine-mediated signaling pathways that regulate the activation of hepatic stellate cells (HSCs) and downstream extracellular matrix (ECM) proteins. These cytokines include TGF-β, platelet-derived growth factor (PDGF), TNF-α, interferons (IFNα/β), and interleukins (IL-1/6/17) [41,42]. Of all the different cytokine mediated pathways, upregulated TGF-B signaling is thought to be the principal fibrogenic pathway that activates HSCs to synthesize fibrogenic materials like collagen and alpha smooth muscle actin (α-SMA) [106,107,108,109]. In HBV-infected patients, the HBx protein has been identified as an activator of cytokine signaling [110], notwithstanding the synergistic effect induced by chronic inflammation, oxidative stress, and hepatocyte loss that triggers the activation of quiescent HSCs into myofibroblasts, which are the main source of ECM production (e.g., collagen 1/111, α-SMA) in the liver [106,107].

**Scheme 4:**
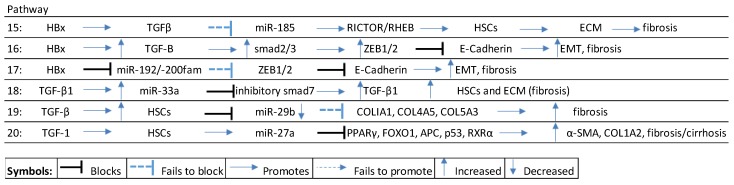
HBx-mediated fibrosis/cirrhosis pathways. TGFβ: transforming growth factor beta; RICTOR: rapamycin-insensitive companion of mammalian target of rapamycin; RHEB: Ras homolog enriched in brain; HSC: hepatocyte stellate cells; ECM: extracellular matrix; ZEB1/2: zinc finger E-box-binding homeobox1/2.

A hypothesized HBx-directed fibrogenic miRNA pathway involves miR-185 as follows: HBx promotes TGFβ signaling, that blocks miR-185 that, in turn, fails to modulate Rapamycin-insensitive companion of mammalian target of rapamycin (RICTOR/RHEB), resulting in the activation of HSCs, ECM proteins, and the development of fibrotic tissue [111,112] (see Scheme 4, pathway 15).

A wide range of miRNA that modulate fibrogenesis are deregulated in HBV-induced liver injury from early stage reversible fibrosis to irreversible fibrosis, loss of liver function and cirrhosis (See Table 2). miRNA directly targeting the TGF-β pathway, for example, include miR-122/-181b/-21/-214-3p/-221-3p/-222/-29/-33a/-942. Typically, HBx-induced TGF-β signaling induces fibrogenesis by sequestering the transcriptional ability of coSmad4 to form a complex to activate smad2/3, thus promoting the transcription of zinc finger E-box-binding homeobox proteins (ZEB1/2), which blocks E-cadherin formation to reduce cell adhesion and promote epithelial mesenchymal transition (EMT) [28,113,114] (see Scheme 4, pathway 16).

Another important miRNA family that modulates fibrogenesis is the miR-200 cluster. In HBV-related fibrogenesis, the HBx protein suppresses p53-led transcription of miR-192/-200, which then fails to modulate ZEB1/2, blocking the transcription of E-cadherin in the WNT/β-catenin pathway. A loss of E-cadherin is a key feature of fibrosis/EMT and occurs early in the HBV-HCC continuum [18,28,115] (see Scheme 4, pathway 17).

HBx-induced TGF signaling in the early phase of liver disease also promotes the increase in ECM, as a result of TGF-β1 upregulation of miR-33a to block inhibitory SMAD7, thus promoting Rsmad-induced TGF-β1, HSCs, ECM, and fibrosis [116] (see Scheme 4, pathway 18).

Collagen is a key downstream fibrogenic product of TGF signaling and cytokines like TFG-β, IL4/13, for instance, trigger TGF-β1-activated smad3, Stat6 to transcribe collagen type 1 alpha chain 2 (COL1A2) [112]. TGF signaling typically employs miRNA to amplify the production of collagen. For example, TGF-β activation of HSCs can downregulate miR-29b that then fails to modulate the expression of collagen [117] (see Scheme 4, pathway 19). TGF-β1-activated HSCs also enhance the expression of miR-27a, which suppresses antagonists of α-SMA and collagen like PPARϒ, FOXOI, APC, p53, and RXRα to promote fibrogenesis [118] (see Scheme 4, pathway 20).

### 2.4. HBV Deregulated MiRNA in HBV-HCC

In this stage, persistent HBV infection, chronic inflammation, oxidative stress, and cirrhosis can dysregulate a wide range of host gene expression by initiating deletions, amplifications, mutations, epigenetic changes, or by targeting miRNA loci or their transcription factors [139]. In this disrupted tissue, HBV-HCC may develop from cells that are able to survive in cirrhotic livers and that are more resistant to adverse conditions, viral infection, and apoptosis [18]. In this regard, the HBx protein is thought to play a key role in the development of HCC because it can inhibit the TP53 function in early carcinogenesis, and may contribute to the accumulation of aberrant replacement cells by downregulating apoptosis [18,140]. The HBx transactivating protein promotes cell cycle progression, inactivates negative growth regulators, tumor suppressors, and senescence-related factors [141,142].

#### 2.4.1. HBV–HBx-Downregulated MiRNA in HBV-HCC

In general, downregulated miRNAs lose their ability to modulate oncoprotein expression. In HBV-infected persons, the HBx protein downregulates a wide range of miRNA (see Table 3) that then fail to modulate host oncogenic target genes and/or epigenetic mechanisms [143,144]. In Table 3, for example, key HBx-downregulated liver miRNA, like miR-122, typically fail to modulate oncogenic cell cycle proteins (cyclins/ β-catenin/ADAM 10 and 17/BCL-W), while Let-7 fails to suppress oncogenic proteins involved in angiogenesis, growth, and migration (STAT3/RAS/C-MYC/BCL-Xl). Another well cited miRNA that is downregulated by the HBx protein is miR-148a, which also fails to suppress oncogenic expression in the P13/MAPK pathway (see Figure 1).

#### 2.4.2. HBx-Upregulated MiRNA in HBV-HCC

In general, many HBx-upregulated miRNAs typically block tumor suppressor networks (see Table 4). For example, the phosphatase and tensin homologue (PTEN) tumor suppressor is downregulated by HBx upregulated miR-21, miR-29a, miR-221, and miR-222. Upregulated miR-21 is a key liver miRNA that has been consistently cited as a silencer of tumor suppressors like PTEN and PDCD4 (see references in Table 4).

## 3. HBV–HBx-Dysregulated MiRNA in the Principal HBV-HCC Cancer Pathways

Understanding of the molecular etiology of HCC remains incomplete [244]. Evidence to date shows that HCC generally involves a range of disruptions of the PI3K/MAPK pathways and the p53 network that includes cell cycle controls (RB1), as well as increased WNT signaling and the inactivation of key tumor suppressors (*SOCSI*) in the JAK/STAT network [18,19]. Alterations of RB1, p53, and WNT pathways in HCC are frequently associated with HCV, HBV, and alcoholic liver cirrhosis [245]. This section focuses on examples of HBx-induced dysregulation of miRNA in HBV-HCC pathways.

### 3.1. Dysregulated MiRNA in the p13K/MAPK Pathway in HBV-HCC

A wide range of HBx-dysregulated miRNA (see Figure 1) play a complex regulatory role in the activation of the PI3K/AKT/mTOR and MAPK (RAF/MEK/ERK) pathways, which are a key feature of HCC [246,247]. Typically, a range of HBx-upregulated miRNA suppress tumor suppressor regulation and HBx-downregulated miRNA fail to modulate oncogenic proteins. Examples of HBx dysregulation include the upregulation of miR-17-92, miR-21 (via IL-6 activation) [55,226,248], miR-29, miR-155-5p [222], and miR-221/-222 [54,231] to suppress *PTEN* regulation of AKT/mTOR expression. Simultaneously, HBx downregulates miR1 (via EDN-1), miR-148a/-34a/26a/c and Let-7, which then fail to modulate MET/HGF [29,147,149,173,197] and RAS/RAF/mTOR expression respectively [145,249]. The subtle HBx counter modulation of mTOR is illustrated by HBx-upregulated miR-7 that increases inhibition of mTOR signaling [241,250], while HBx-downregulated Let-7 fails to modulate mTOR signaling. Simultaneously, HBx-induced downregulation of miR-1 can promote HBV replication via HDAC4-mediated stimulation of FXRα. HBx also downregulates miR-29c/-125b, resulting in reduced controls for the transcription of BCL-2 [28,200,201] and miR-26a/-34a that modulate C-JUN/FOS/cyclinD expression [28,197].

### 3.2. Dysregulated MiRNA in the WNT/β-Catenin Pathway in HBV-HCC

The upregulation of the WNT/β-catenin pathway is a frequent event in early HCC [251]. It yields an aggressive phenotype that is implicated in the proliferation, migration, invasion, and survival of cancer cells [244]. Figure 2 illustrates some examples of HBx-dysregulated miRNAs and their target genes in this pathway. The HBx protein typically plays a role in enhancing WNT and β-catenin expression while suppressing the expression of E-Cadherin. WNT signaling, for instance, is not modulated as a result of HBx led downregulation of miR-122/148a/b [157,173,174] and Cadherin expression is suppressed because these miRNA fail to suppress cadherin suppressors like SNAIL 1 [174]. HBx-induced upregulation of mIR-21 also enhances WNT signaling because it reduces DCC6 suppression of WNT signaling [252] and contributes to the suppression of E-cadherin by suppressing *PDCD4* expression [55]. HBx-upregulated miR-221/-222 also contributes to the suppression of cadherin by enhancing *ZEB 1/2* via blocking one of its suppressors like *TRPS-1* [54,157]. Cadherin expression is also suppressed as a result of HBx-induced downregulation of miR-200/-205/-101/34 that fails to regulate ZEB 1/2 [95,150,190]. HBx-induced downregulation of miR-122 reduces β-catenin-led transcription in the cytoplasm [156], while HBx upregulation of miR-155 blocks the *APC* tumor suppressor in the WNT pathway, as well as suppresses HBV replication by blocking enhancer 11 to potentially hide the virus from the host immune system [28,31].

### 3.3. Dysregulated MiRNA in the TP 53 Pathway in HBV-HCC

The deregulation of multiple p53 pathways is a central event in the progression of HBV-HCC [253]. The *INK4alpha/ARF* locus, that encodes p14(ARF) and p16(INK4alpha) to arrest the cell cycle in the p53 and RB pathways, is frequently disrupted in HCC [254]. Figure 3 illustrates how HBx directly targets p53 expression [28,241] to influence the expression of miRNAs in this pathway. Typically, the HBx protein downregulates miR-26a/-34a/-138/15a/16-1, that fail to modulate cyclinD/CDK 4/6 and cyclinE/CDK2 expression [78,168,197,207], as well as miR-122/-Let-7/-34a/-125b/15a/16-1 [78,145,255,256,257,258], that then fail to modulate BCL-2-mediated suppression of CASP9/3-induced apoptosis. Similarly, HBx downregulation of miR-101/-125b/-29a/-Let-7 exerts an anti-apoptotic effect by failing to modulate MCL-1-mediated suppression of CASP9/3-induced apoptosis [200,255,258,259]. HBx-induced C-MYC upregulation of miR-17-92 and miR-221/222 also contribute to the E2F-mediated downregulation of *p21/p27/p57* expression as a regulator of cyclins and cyclin-dependent kinases (CDKs) in the network [82,223,230].

### 3.4. Dysregulated MiRNAs in the JAK/STAT Pathway in HBV-HCC

The aberrant methylation in the CpG island of the SOCS-1 gene is a common feature in HBV-HCC and its silencing demonstrates its important tumor suppressor role in the JAK/STAT pathway [260]. Suppressor of cytokine signaling (SOCS-1) switches this signaling ‘off’ by means of its direct interaction with (JAK). The loss of function of SOCS-1 is a common feature in HCC and the HBx-mediated upregulation of miR-155 is a contributing factor in HBV-HCC (see Figure 4) [261,262]. HBx upregulated miR-221/-203 contribute to a reduction in SOCS3 regulation of JAK/STAT signaling [263]. HBx also downregulates Let-7, which reduces its modulation of IL-6 induced activation of JAK/STAT signaling and mTOR mediated transcription of oncogenic proteins like C-MYC/MCL-1 [145,250,264]. However, HBx upregulation of miR-7 [241], has been demonstrated as a control by way of suppressing mTOR signaling in JAK/STAT pathway [250].

## 4. Discussion and Conclusions

This exploratory review deliberately adopts a broad focus to demonstrate the complex regulatory role of miRNAs in the HBV-HCC continuum. Multiple knowledge gaps, exposed in this paper, prompt further research, to clarify the complex regulatory roles of the same miRNA across the HBV-HCC continuum. In addition, numerous miRNA target the same genes and cascades of miRNA respond to injury and disease with differing levels of expression in tissue, serum and cell-lines. Circulating miRNA, for instance, may originate from different cells (e.g., blood-immune) rather than an HBV or cancer specific origin by way of secretion or cell death [265,266,267]. The broad focus of the paper is, therefore, specifically adopted to highlight this complexity rather than to explain it. The key focus of this exploratory review was, therefore, to illustrate the multiplicity of dysregulated miRNA in clearly defined stages of pathogenesis rather than to attempt to try and explain their role in mediating multiple targets or summarize their role across every stage of HCC pathogenesis (see miR-122/-21). A key limitation of the study, therefore, is that it conveys an overly simplistic role of specific miRNA in HBV-HCC pathogenesis.

The potential roles of HBx downregulated miR-124, illustrates this limitation (see Table 3). Its various roles could include interaction with lncRNA-MALAT1 to regulate HBx-induced cancer stem cell properties in HepG2 through PI3K/AKT signaling [268]. miR-124 also suppresses cell proliferation and tumor growth in HCC in vitro and in vivo models by direct targeting *STAT3* and *PIK3CA* thereby repressing both JAK/STAT and PI3K/AKT pathways [164]. Other studies demonstrate that miR-124 can suppress cell proliferation in HCC by targeting *PIK3CA* [269], as well as extracellular matrix protein laminin gamma 1 (LAMC1), which is a key feature in HCC progression [270]. However, *LAMC1* mRNA promote malignancy by competing with miR-124 by binding with CD151 [270] and the overexpression of LAMC1 promotes HCC progression pathway by interaction with integrin receptors on a cell surface to promote proliferation and metastasis in HCC [271]. Elevated *LAMC1* mRNA also acts as a sponge for miR-124 thus preventing its binding to another oncogenic membrane protein (CD151) that contributes to its elevated expression in HCC [270].

Similarly, the figures illustrating isolated roles of HBx-dysregulated miRNA in the main HBV-HCC pathways are clearly simplistic and only serve to demonstrate the interaction of the HBV dysregulated miRNA and their respective targets, thus ignoring the fact that they only represent a fraction of miRNA targeting these cancer pathways. The present review illustrates the complex range of miRNA regulatory roles in HBV-HCC pathogenesis. It also demonstrates how miRNA manipulation of HBV expression can be used as a tool to dissect HCC molecular pathways, and be harnessed to improve diagnosis, prognosis, anti-viral, and anti-tumor therapeutic modalities.

## Figures and Tables

**Table 1:** Dysregulated miRNA in the HBV infection/inflammation stage (in miR alpha-numeric order).

miRNA	HBV	Comment	Author
Let-7 fam	Down	Let 7a/b/c/d/e/f/g/I all downregulated by HBx protein which inhibits *IL-6, IL-10, TLR4* expression	[40,51]
Let-7a-3p	Up	Upregulated in early stage HBV/targets *DDX3X*/expression can be downregulated in sera but upregulated in tissue	[57,58]
Let-7c	Up	Impairs immune response and HBV evasion/targets *HDAC4/MET* to enhance HBV replication via *FXRA*	[58,59,60]
miR-1	Up	Targets *HDAC4, MET* to enhance (HBV replication)HBV RE by augmenting *FXRA* expression	[56]
miR-10a	Up	Upregulated > three-fold in HBV infection/upregulated in sera, Targets *EphA4* which is an epithelial-mesynchymal-transition (EMT) suppressor	[58,59,61]
miR-106a	Down	Downregulated by HBx, targets *IL-8* in CHB	[40,62]
miR-122	Up	Upregulated in sera for HBeAg positive patients/correlates with HBV DNA/in sera and tissue	[32,60,63,64]
	Up	Upregulated in CHB vs. Healthy Controls (HC)/upregulated in CHB vs. HC sera	[25,33,59,65]
	Up	Upregulated in HBV also HBeAg+ vs. HBeAg–/correlates with HBV DNA/HBsAg	[63,66]
mir-122	Down	Suppress HBV by targeting HBV mRNAs/targets *Cyclin G1/HO-1* to promote HBV infection by inhibiting *HO-1*	[28,35,36,67,68]
miR-122-5p	Up	Upregulated in CHB/biomarker for liver injury	[69]
	Down	Decreased level in occult HBV infection (OBI) vs. HC	[70]
miR-124	Up	Serum miRNA correlates with necrosis/inflammation and upregulated in CHB vs. HC/targets *AQP3*	[71,72]
miR-125	Up	Impairs immune response and HBV evasion	[56]
miR-125a-5p	Up	Correlates with HBsAg/HBeAg and HBV-DNA/suppresses HBV expression by blocking HBsAg mRNA/targets *KLF13*	[40,58,73,74]
miR-125a	Up	HBx upregulated in HBV infection	[40,75]
miR-125b	Up	Upregulated in CHB sera/correlated with HBV DNA, HBsAg, HBeAg	[59,63]
miR-125b-5p	Up	Upregulated 2.85 fold in CHB/targets *STAT3*	[57,58,76]
miR-1275	Up	Upregulated in HBV patients/correlated with serum ϒ-glutamyl transpeptidase	[63]
miR-130a	Up Down	Upregulated by NFκB/ Marker for OBI/upregulated vs. HC, ASC, CHB Targets *PGC1α* and *PPARγ* to regulate HBV replication/possible dual role in HBV	[70] [77]
miR-15a	Down	Downregulated by HBx/Suppresses HBV infection by binding to HBx mRNA/targets *TGF-β/Smad7*	[28,78,79]
miR-146	Up	Targets/stimulates HBx/NFκB in HBV infection	[80]
miR-150	Up	Upregulated > three-fold in HBV sera/downregulated in HBV asymptomatic vs. CHB	[59,60,81]
miR-151-3p	Up	Upregulated in CHB/biomarker for liver injury	[69]
miR-155	Up	Blocks S*OCS1* to upregulate JAK/STAT/targets *C/EBP* Suppresses HBV by augmenting INF signaling	[30,31]
miR-16-1	Down	Downregulated by HBx RNA	[78]
miR-17	Down	Downregulated in HBV-infected persons	[40]
miR-17-92	Up	HBV transactivates *c-Myc* to upregulate miR-17-92 which suppresses HBV mRNA	[82]
miR-181a-d	Up	Correlated with HBV DNA and HBV disease progression/downregulates HLA-A, HBsAg by targeting *HLA-A, E2F5*	[40,83,84,85,86]
miR-182	Down	Downregulated in CHB leading to HBV-related cirrhosis	[87]
miR-191	Up	Upregulated by HBx protein	[40]
miR-192	Up	Upregulated in CHB vs. HC serum	[65,88]
miR-192-5p	Up	HBV activated/correlated with HBV Replication	[88,89]
miR-193a-5p	Down	Downregulated in A3 vs. A0 inflammation/targets *SPOCK3*	[90,91]
miR-193b	Up	Upregulated by HBx protein/directly targets *Mcl-1*	[40,92]
miR-196a	Down	Downregulated by HBx	[40,83,93]
miR-196c	Up	Upregulated in HBV-infected persons	[40]
miR-1974	Up	Upregulated in CHB vs. normal liver (NL)/upregulated A3 vs. A0 inflammation	[90]
miR-199a-5p	Up	Upregulated in early HBV-immune tolerant/HBx upregulated/Target *STAT2*/low in blood sera but up in tissue	[40,57]
miR-20a	Down	Downregulated by HBx	[40]
miR-200b	Down	Downregulated in sera (ECVs) in CHB	[81]
miR-200c	Down	HBx reduced in HBV infection/targets nuclear factor IA to suppress HBV replication	[28,94]
miR-205	Down	Targets HBx mRNA	[28,95]
miR-203a	Up	Upregulated in HBV infection/promotes inflammation targets *IL-6, IL-8*	[96]
miR-21	Up	Upregulated in A3 vs. A0 inflammation/impairs immune response and HBV evasion/up in CHB vs. HC serum	[56,65,90]
miR-210	Up	Correlates with HBV DNA and HBsAg/marker for necroinflammation/suppresses HBV infection by targeting HBsAg pre-S1 region	[58,59,97,98]
miR-215-5p	Up	HBV activated miRNA/correlates with HBV replication	[89]
miR-221-3p	Up	Upregulated in early HBV/targets *TBK1*/upregulated in blood sera/tissue	[57]
miR-22	Up	Upregulated in HBV-infected persons/correlates with HBeAg, HBeAg positive persons/correlated with miR-122	[32,63]
	Up	Upregulated in A0 vs. A3 inflammation/targets *HDAC4, ERα* to inhibit HBsAg/HBeAg	[90]
miR-223-3p	Up	5.55-fold upregulated in CHB in blood sera	[59,76]
miR-23a/b	Up	Upregulated > three-fold in HBV infection/upregulated in sera	[40,58,59,83]
miR-236	Up	Upregulated in CHB vs. HC sera	[59]
miR-30c	Up	Upregulated by HBx protein	[40]
miR-3200	Up	Upregulated in CHB leading to LC	[87]
HBV-mir-3	Up	Mediates HBV RE by blocking HBc mRNA to downregulate HBV virions	[27]
miR-338-3p	Down	Downregulated in HBV	[40]
miR-339	Up	Upregulated in CHB leading to LC	[87]
miR-34a	Up	Upregulated A3 vs. A0 inflammation/promotes Tregs that block effector T-cells	[28,29,90]
miR-342-3p	Up	Upregulated > three-fold in HBV infection in sera/HBx-induced deregulation	[40,58,59,87]
miR-3613-3p	Up	Upregulated in CHB/Target *STAT3*/down in sera	[57]
miR-3615	Up	Upregulated in CHB leading to LC	[87]
miR-371	Up	Upregulated by HBx protein	[40]
miR-372	Up	Correlates with HBV DNA in CHB/deregulated by HBx/targets *NFIB/HNF4, RXR,* and *PPAR* to regulate HBV transcription	[39,40,99]
miR-373	Up	Correlates with HBV DNA in CHB/deregulated by HBx	[39,40]
miR-375	Up	Upregulated > three-fold in HBV in sera	[58,59]
miR-378	Down	Down-regulated in HBV	[40]
miR-423	Up	Upregulated > three-fold in HBV infection/upregulated in sera	[58,59]
miR-4485	Down	Downregulated in CHB re progression to HBV-LC	[87]
miR-451	Up	Upregulated in CHB vs. NL/Upregulated in A3 vs. A0 inflammation	[90]
miR-4508	Up	Upregulated in CHB leading to LC	[87]
miR-4717	Down	Significant upregulation of *PD-1* (programmed cell death)	[100]
miR-486-5p	Down	Downregulated in CHB vs. NL	[90]
MiR-501	Up	Targets HBX1P to induce HBV RE	[101]
miR-548	Down	Inhibits *IFN-ϓ1*, downregulates immune response/promotes HBV	[102]
miR-548d-5p	Up	Upregulated in A3 vs. A0 inflammation	[90]
miR-602	Up	Upregulated in CHB vs. HC/targets *RASSFIA*/ascending upregulation on HBV-HCC continuum	[103]
miR-659	Down	Downregulated in A3 vs. A0 inflammation	[90]
miR-711	Down	Downregulated in A3 vs. A0 inflammation	[90]
miR-720	Up	Upregulated in HBV patients also correlates with HBeAg+ HBeAg–	[63]
miR-760	Up	Upregulated in A3 vs. A0 inflammation	[90]
miR-762	Down	Downregulated in CHB vs. NL	[90]
miR-767-3p	Up	Upregulated in A3 vs. A0 inflammation	[90]
miR-885-5p	Up	Upregulated in CHB vs. HC	[104]
miR-92a	Up	Upregulated > three-fold in HBV in sera/	[58,59,81]
miR-935	Up	Upregulated in CHB leading to LC	[87]
miR-940	Down	Downregulated in CHB/downregulated in sera and tissue	[57]
miR-9-5p	Down	Downregulated in CHB/even lower with higher fibrosis scores	[105]
miR-99a	Up	Upregulated in HBV+ patients also HBeAg+/upregulated in sera	[58,59,63]
mIr-99b	Up	Upregulated by HBx protein	[40]

NOTE: A0–A3 (low to severe liver inflammation); IL: interleukin; TLR: toll-like receptor; DDX3X: DEAD-Box Helicase 3 X-Linked; HDAC4: Histone Deacetylase 4; FXRA: farnesoid X receptor A; CHB: chronic hepatitis B: HC: healthy control; ASC: asymptomatic carrier; LC: cirrhosis; NL: normal liver.

**Table 2:** Dysregulated miRNA in HBV-associated liver fibrosis/cirrhosis (in miR numeric order).

MiRNA	Deregulation	Comment	Author
miR-1	Up	Upregulated in late-stage fibrosis (f3–4)/targets *ATF2/E2F3/CREB3L2*/up in tissue and sera	[57]
miR-10b-5p	Up	Upregulated advanced fibrosis vs. early fibrosis/targets *ATF2/E2F3/CREB3L2*/up in tissue and sera	[57]
miR-101	Down	Downregulated in HBV fibrosis/HBx downregulated via *EZH2*-induced methylation/suppresses *TGF-B* signaling to block collagen	[58,119]
	Up	Suppresses *TGF-β* signaling to block collagen/upregulated in cirrhosis versus CHB	[120]
miR-103	Up	Upregulated in cirrhosis vs. CHB	[118]
miR-122	Up	Upregulated in early fibrosis/significant decrease as f0–2 progresses to f3–4	[33,58,81,121]
	Down	Downregulated in advanced f4 vs. f0–1/suppresses *TGF-β* pathway to block collagen	[33]
miR-122-5p	Up	Correlates with degree of fibrogenic damage	[69,122,123]
miR-1224-3p	Up	Upregulated in early fibrosis (f1–2)/target *ELK1/AKT2*/upregulated in sera/tissue	[57]
miR-1227-3p	Up	Upregulated early fibrosis (f1–2)/target *HSPG2/PTEN*/downregulated in sera	[57]
miR-125b-5p	Up	Upregulated in cirrhosis	[76]
miR-125a-5p	Up	Targets *F1H1* in HBV-related fibrosis vs. H. Controls	[124,125]
miR-126	Up	Upregulated in cirrhosis vs. CHB stage	[118]
miR-1275	Down	Downregulated in cirrhosis vs. CHB stage	[118]
miR-128	Up	Upregulated in cirrhosis vs. CHB	[118]
miR-130a	Up	Upregulated in fibrosis/f3 > f0/upregulated in cirrhosis vs. CHB/IFNs trigger *JAK/STAT*	[118]
miR-133a	Down	Downregulated in cirrhosis	[126]
miR-133b	Up	Upregulated in advanced vs. early fibrosis/targets *ATF2/E2F3/CREB3L2*/upregulated in sera and tissue	[57]
miR-140-5p	Up	Upregulated in cirrhosis vs. CHB	[118]
miR-141-3p	Down	Downregulated in cirrhosis vs. HCC	[123]
miR-142-3p	Up	Upregulated in cirrhosis vs. CHB	[118]
miR-143	Down	Downregulated in advanced fibrosis vs. early fibrosis	[127]
miR-146a	Up	Upregulated in CHB fibrosis/upregulated in cirrhosis vs. HC/HBx upregulated > CFH fiber proteins	[104,118,121,128]
miR-148a/b	Up	Upregulated in cirrhosis vs. CHB	[118]
miR-149-5p	Down	Downregulated in HBV-induced cirrhosis	[129]
miR-150	Down	Inhibits collagen-1 expression and HSC activation/targets c-myb	[130]
miR-151a-3p	Down	Downregulated as injury increases	[69]
miR-151-5p	Up	Upregulated in cirrhosis vs. CHB/predictor of activated HSCs	[118]
miR-17	Up	Upregulated in cirrhosis vs. CHB	[118]
miR-17-3p	Down	Downregulated as injury increases/downregulated in cirrhosis vs. fibrosis	[129]
miR-17-92	Down	MiR-17-92 family (miR-19a/b/92) downregulated in activated HSCs	[131]
miR-181b	Up	Promote fibrosis via *TGF-β or NF-κB* pathways/correlates with HBV DNA/downregulated in f4 vs. f1/upregulated in cirrhosis	[58,126]
miR-185	Down	TGF-1 > miR-185 > *RICTOR/RHEB* > HSCs > ECM > fibrosis/downregulated in HBV-induced fibrosis	[111]
miR-1915	Down	Downregulated in cirrhosis vs. CHB	[118]
miR-19b	Down	Downregulated in activated HSCs/CHB led fibrosis/f3 > f0	[131]
miR-192	Down	Downregulated in sera of CHB patients re ECVs	[81]
miR-193-5p	Down	Downregulated in cirrhosis vs. HBV-HCC	[123]
miR-194	Down	Inhibits collagen-1 expression and HSC/targets *rac1*	[130]
	Up	Upregulated as injury increases/targets *ACVR28*	[132]
miR-199	Down	Downregulated as injury/cirrhosis develops	[126]
miR-199-5p	Up	Upregulated in cirrhosis vs. CHB	[118]
miR-199a-3p	Down	Upregulated in cirrhosis vs. CHB	[118]
miR-199a	Up	Upregulated in HBV-led fibrosis	[40]
miR-200a/b	Up	Upregulated in early HBV-fibrosis	[40,81]
miR-20a	Up	Upregulated in CHB fibrosis/f3–4 < f0–2	[121]
miR-20b-5p	Down	Downregulated in advanced fibrosis versus early fibrosis/Targets *p21*/upregulated in sera	[57]
miR-206	Down	Downregulated in cirrhosis vs. HBV-HCC	[123]
miR-21	Down	Downregulated in advanced fibrosis vs. early fibrosis/acts via *TGF-B/NF-kB* pathways/downregulated f0–2 > f3–4 in CHB fibrosis	[58,118,127]
	Down	Downregulated in cirrhosis progression	[118,133]
miR-214-3p	Down	AUC 0.87 predictor of fibrosis/suppresses TGF-B pathway blocks collagen	[58,134]
miR-214-5p	Up	Upregulated in cirrhosis	[126]
miR-215	Up	Upregulated in cirrhosis vs. HC	[104,128]
miR-221-3p	Up	Promotes fibrosis via *TGF-B/NF-kB* paths/f4 down vs. F1	[57,118]
miR-221	Down	Downregulated in CHB fibrosis but/f3–4 < f0–2	[58,121]
	Up	Upregulated in cirrhosis	[118,126]
miR-222	Up	CHB fibrosis via *TGF-β/NF-κB* pathways/increasing significant upregulation from f0–4Upregulation in cirrhosis	[58,121,126]
miR-222-3p	Up	Upregulated 13.88-fold in cirrhosis	[76,118]
miR-223	Down	Downregulated in advanced fibrosis vs. early fibrosis/see article for path	[127]
miR-224	Down	Downregulated in CHB led fibrosis/f3–4 significantly less downregulated than f0–2	[121]
	Up	Upregulated in cirrhosis vs. HC	[104,128]
miR-23a/b	Up	Upregulated in cirrhosis vs. CHB	[118]
miR-26a	Down	Increasingly downregulated in cirrhosis continuum	[133]
miR-26a-5p	Up	Upregulated in cirrhosis vs. HBV-HCC	[123]
miR-27-3p	Up/Down	Biomarker for cirrhosis	[129]
miR-27a	Up	TGF-1 > miR-27a > *PPARγ, FOXO1, APC, P53* and *RXRα α-SMA* and *COL1A2* (activated HSCs)	[118]
miR-27b	Up	Upregulated in cirrhosis vs. CHB	[118]
miR-2861	Up	Upregulated stage f4 vs. f0 fibrosis	[135]
miR-29a	Up	Upregulated in CHB fibrosis/f3–4 < f0–2	[121]
	Down	Increasingly downregulated as cirrhosis develops via *TGF-B, NFκB*	[40]
miR-29b	Down	Blocks collagen/TGF-B/targets *COL1A1, COL4A5, COL5A3*	[58,117]
miR-30b/c	Up	Upregulated in cirrhosis vs. CHB	[118]
miR-30e	Down	HBx-led downregulation of miR-30e >> *P4HA2* fibrosis/cirrhosis	[118,123,136]
	Down	Targets *IL-6R* in fibrogenic pathway	[134]
miR-301a	Up	Upregulated in cirrhosis vs. CHB	[118]
miR-324-5p	Up	Upregulated in cirrhosis vs. CHB	[118]
miR-33a	Up	Stimulates *TGF-B*, HSCs and promotes fibrosis/targets *SMAD7* to stimulate *TGF-B* HSCs/upregulated in cirrhosis vs. CHB	[116,118]
miR-331-3p	Up	Upregulated in cirrhosis vs. CHB	[118]
miR-338-3p	Up	Upregulated in cirrhosis vs. CHB	[118]
miR-34a/b/c	Up	Upregulated in HBV-led fibrosis	[40]
miR-34b-3p	Up	Upregulated in early fibrosis/targets *GRB10/PIK3CA*/up in sera/tissue	[57]
miR-340	Up	Upregulated in cirrhosis vs. CHB	[118]
miR-345-3p	Up	Upregulated in stage f4 vs. f0 fibrosis	[135]
miR-346-3p	up	Upregulated in early CHB induced fibrosis stages f1–2	[57]
miR-3620-3p	Up	Upregulated in stage f4 vs. f0 fibrosis/cirrhosis	[135]
miR-3656	Up	upregulated stage f4 vs. f0 fibrosis/cirrhosis	[135]
miR-371a-5p	Up	Upregulated in stage f4 vs. f0 fibrosis/cirrhosis	[135]
miR-374	Down	Downregulated in advanced fibrosis vs. early fibrosis	[127]
miR-374b	Up	Upregulated in cirrhosis vs. CHB	[118]
miR-377-3p	Up	Biomarker for cirrhosis	[129]
miR-410-3p	Up	Biomarker for cirrhosis	[129]
miR-424	Up	Upregulated in cirrhosis vs. CHB	[118]
miR-455-3p	Down	Downregulated in advanced fibrosis vs. early fibrosis/Targets *p21*/upregulated in sera	[57]
miR-4646-5p	Up	Upregulated f4 vs. f0 fibrosis/cirrhosis	[135]
miR-4651	Up	Upregulated f4 vs. f0 fibrosis/cirrhosis	[135]
miR-4695-5p	Up	Upregulated f4 vs. f0 fibrosis/cirrhosis	[135]
miR-4800-5p	Up	Upregulation f4 vs. f0 fibrosis/cirrhosis	[135]
miR-483-5p	Down	Downregulated in f3 vs. f0 fibrosis/cirrhosis	[90]
miR-486-3p	Down	Downregulation f4 vs. f0 fibrosis/cirrhosis	[135]
miR-486-5p	Down	Downregulated in cirrhosis vs. HBV-HCC	[123]
miR-497-5p	Down	Downregulation f4 vs. f0 fibrosis/cirrhosis	[135]
miR-499a-5p	Down	Downregulated in early HBV-induced fibrosis/target *CDKNIA/IKBKB*/upregulated in sera	[57]
miR-513-3p	Up	Upregulated in early cirrhosis	[58,118]
miR-571	Up	Upregulated in early cirrhosis	[58,118]
miR-574-3p	Up	Upregulated in cirrhosis vs. HC	[104,128]
		Upregulated early cirrhosis/differentiates between cirrhosis and HBV-HCC	[118]
miR-602	Up	Upregulated in cirrhosis vs. HC/increasing in HBV-HCC	[103,128]
miR-615-3p	Up	Promotes hypersplenism/cirrhosis	[137]
miR-638	Up	Upregulated f4 vs. f0 fibrosis/cirrhosis	[135]
miR-652	Up	Upregulated in cirrhosis vs. CHB	[118]
miR-671-5p	Down	Downregulated in advanced versus early fibrosis/targets *ATF2/E2F3/CREB3L2*/up in sera and tissue	[57]
miR-744	Up	Upregulated in cirrhosis vs. CHB	[118]
miR-885-5p	Up	Upregulated in cirrhosis versus HC	[104,128]
miR-92	Down	Downregulated in CHB fibrosis/f3–f4 > f0–f2	[121]
miR-939	Down	Downregulated in cirrhosis vs. CHB	[118]
miR-940	Down	Downregulated in cirrhosis vs. CHB	[129]
miR-942	Up	Upregulated in activated HSCs/TGF and LPS induced miR-942	[138]
miR-9-5p	Down	Fibrosis/activates HSCs via *TGFBR1/TGFBR2*	[105]
miR-96-5p	Up	Upregulated in advanced fibrosis vs. early fibrosis/targets *ATF2/E2F3/CREB3L2*/up in sera and tissue	[57]

NOTE: f0–4 (fibrosis stage levels).

**Table 3:** HBV–HBx-downregulated miRNA in HBV-HCC (in alpha-numeric order).

miRNA	Expression	Target	Gene Function	Reference
let-7 fam	Down	*STAT3/RAS/HMGA2C-MYC/IL-6/IL-10/TLR-4/COL1A2/NGF, BCL-XL*	Angiogenesis/growth/migration/inflammation/HBx repression	[51,52,145,146]
miR-1	Down	*EDN1/PI3K/AKT/HDAC4/MET*	Angiogenesis, migration, invasion/modulates HBV replication	[147,148,149]
miR-101	Down	*DNMT3A/RASSF1/PRDM2/GSTP1/FOS/MCL-1/EZH2*	Proliferation/migration/invasion, increases HBV replication/induce DNA methylation	[120,150,151,152,153]
miR-101-3p	Down	*ND, RAP1B/MCL-1,SOX9*	Inhibits HBV, proliferation/migration/promotes apoptosis	[154,155]
miR-122	Down	*β-CATENIN*	EMT, cell migration, invasion and metastasis. Downregulated by HBx DNA methylation	[28,156]
	Down	*CCNG1* modulated *p53/GLD2*	Promotes cell cycle progression/increases HBV replication	[35,157,158,159]
	Down	*NDRG3/GALNT10 Cyclin G1/PTTG1*	Promotes apoptosis/blocks cell proliferation/invasion	[68,143,160,161]
	Down	*PBF/ADAM10/Cyclin G1/Igf1R/ADAM* 17/*BCL-W/NDRG3*	Modulates proliferation, invasion, apoptosis	[28,35,68,162,163]
miR -124	Down	*STAT3 and PIK3CA*	Suppresses cell proliferation	[164]
miR-125b	Down	*SMAD2/4*/*SIRTUIN7*/*SUV39H1*/*LIN28B/PIGF*	Modulates EMT, growth, migration, and invasion	[28,165]
miR-132	Down	*AKT*	Enhances cell proliferation/HBx DNA methylation downregulates miR-132	[166]
miR-136	Down	*AEG-1*	Cell migration, invasion, and metastasis	[167]
miR-138	Down	*CCND3*	Promotes cell cycle progression/reduces HBV replication	[168]
miR-139-5p	Down	*ZEB1/2*	Modulates EMT/metastasis	[28,169,170]
miR-145	Down	*MAP3K/CUL5/HDAC2/ADAM17*	Enhances proliferation, cell cycle progression, anti-apoptosis	[54,171,172]
miR-148a	Down	*HPIP/AKT/ERK/FOXO4/ATF5/mTOR/MET/ACVR1*	Down in HCC tissue/Cell proliferation, EMT, and cell migration	[28,173,174,175]
miR-152	Down	*DNMT1/GSTP/CDH1/KIT*	Modulates DNA methylation/cell proliferation/migration/invasion, blocks HBV	[176,177,178,179]
miR-15a/16-1	Down	*CCND1/BCL-2*	Promotes cell cycle progression/proliferation	[28,78,180,181]
miR-15b	Down	*FUT2/GloboH/HNFα*	Enhances cell proliferation	[182,183]
mIR-16	Down	*Cyclin D1, NCOR2*	Promotes apoptosis, decreases proliferation	[28,180]
miR-18a	Down	ERα/CTGF	Enhances proliferation, regulates connective tissue growth	[184,185]
miR-192	Down	*G1-G2* arrest*/SLC39A6/SNAIL*	Cell cycle control/regulates metastasis	[28,186,187]
miR-193b	Down	*ING5/CCND1/ETS1*	Regulate CDK2, proliferation, invasion	[188,189]
miR-200 fam	Down	*ZEB1/2*	Modulates ZEB1/2 regulated E-cadherin	[28,190]
miR-205	Down	*ACSL4/E2F1/ZEB1/2*	Lipogenesis and cell proliferation/promotes EMT	[28,95,191]
miR-21	Down	*PTEN/PIP3/AKT*	Down in serum/inhibits cell proliferation	[54,192]
miR-216b	Down	*IGF2BP2/IGF2/AKT/mTOR/MAPK/ERK*	Cell proliferation and cell migration	[54,193]
miR-222	Down	*p27*	Inhibits cell cycle progression	[54]
miR-23a	Down	*Myc/E-CADHERIN/SPROUTY2*	Regulates EMT/metastasis	[28,194,195,196]
miR-26a/c	Down	*IL-6/IFNα/ERα/IL-6/Cyclin D2/Cyclin E2*	Inhibits cell proliferation and metastasis	[197,198,199]
miR-29c	Down	*BCL-2/MCL-1/TNFA1P3*	Inhibits cell proliferation and promotes apoptosis	[200,201]
miR-338-3p	Down	*CCND1*	Promotes cell cycle progression	[202,203]
miR-34a	Down	*CCL22/MAP4K4/SIRT1/CCND1/CDK4/6/MET*	Modulates metastasis/growth/apoptosis	[29,204,205,206,207]
miR-363-3p	Down	*SP1*	Modulates tumor growth	[28,208]
miR-373	Down	*E-CADHERIN*	EMT, cell migration, invasion, and metastasis	[49]
miR-375	Down	*AEG-1*	Cell migration, invasion, and metastasis	[167]
miR-429	Down	*RAB18, NOTCH1*	Lipogenesis/proliferation, apoptosis	[209,210]
miR-520b	Down	*HBXIP*	Enhancing cell proliferation	[211]
miR-548p	Down	*HBXIP*, IFN-λ1	Increases growth, blocks apoptosis/immune response	[102,212]
miR-661	Down	*MTA1/NK-κB/iNOS/NO*	Angiogenesis, cell proliferation, and migration	[213]

**Table 4:** HBV–HBx-upregulated miRNA in HBV-HCC (in miR numeric order).

miRNA	Expression	Target	Gene Function	Reference
miR-1	Up	*MASPIN*	HBx-induced HBV-HCC progression	[214]
miR-107	Up	*AXIN2/MASPIN*	Cell proliferation/HBV-HCC progression	[214,215]
miR-125a	Up	*ERBB2, HBsAg*	Suppresses HBsAg	[74,178,216]
miR-143	Up	*FNDC3B*	Cell migration, invasion and metastasis	[28,217]
miR-146a	Up	*CFH/STAT1*	Regulates inflammation/IFNα mediated anti- HBV efficiency	[104,218,219]
miR-155	Up	*PTEN/SOX6/ZHX2/SOCS1*	Promotes cell growth	[220,221,222]
miR-17-92	Up	*E2F1 (C-MYC-repressor), Cyclin G1*	Promotes HCC, blocks HBV replication, enhances proliferation and anchorage-independent growth	[82,223,224]
miR-181a	Up	*FAS, E2F5*	Inhibits apoptosis, promotes cell growth	[85,86]
miR-199a-5p	Up	*CHC*	Promoting cell proliferation	[168]
miR-203a	Up	*RAP1A*	Increases inflammation/alters MAPK signaling	[96]
miR-21	Up	*PDCD4, PTEN*	Up in HCC tissue/Targets tumor suppressors/Stops apoptosis/immune response/HBV evasion/promotes cell growth/up in HBV-HCC vs. CHB-L Cirrhosis serum	[54,55,56,65,214,225,226]
miR-215	Up	*PTPRP*	Proliferation of hepatoma cells	[104,186,227]
miR-221	Up	*ERα, DDIT4/BMF/p27/p57*	Cell cycle progression (G1/S) and cell proliferation	[228,229,230]
miR-222	Up	*P27(kip1)/PTEN/PPP2R2A/p27/p57*	Promotes cell growth, migration	[54,230,231,232]
miR-224	Up	*PAK4/MMP9* inhibitor*-5/SMAD4*	Enhance HBV replication, cell growth and invasion	[233,234,235]
miR-27a	Up	PPARγ, FOXO1, APC, P53 and RXRα	Enhances proliferation, migration, invasion	[118,236]
miR-29a	Up	*PTEN/PI3K/AKT/MMP-2*	Enhancing cell migration	[54,237]
miR-30c	Up	*HMBOX1*	Induces NK activity	[146,238]
miR-331-3p	Up	*ING5*	Cell proliferation and apoptosis	[239]
miR-545/374a	Up	*ESRRG*	Cell proliferation and cell migration	[240,241]
miR-602	Up	*RASSF1a/STAT3/MYC*	Cell apoptosis and proliferation, increases HBV	[13,103]
miR-7	Up	*EGFR/RAF/EKERK/PI3K-AKT/MASPIN*	Inhibits cell growth	[214,241,242,243]
